# Defining the Critical Role of α-Gustducin for NF-κB Inhibition and Anti-Inflammatory Signal Transduction by Bitter Agonists in Lung Epithelium

**DOI:** 10.3390/ijms27020997

**Published:** 2026-01-19

**Authors:** Yuzhen Fang, Qiujie Wang, Shuobin Wu, Xinxiu He, Shengyu Wang, Ruonan Ma, Hao Zhao, Xiaoyi Zhao, Xing Wang, Yuxin Zhang

**Affiliations:** 1 Key Laboratory of Ethnomedicine in Ministry of Education, School of Pharmacy, Minzu University of China, Beijing 100081, China; fangyuzhen@muc.edu.cn (Y.F.); wangqiujie@muc.edu.cn (Q.W.); wushuobin@muc.edu.cn (S.W.); hexinxiu@muc.edu.cn (X.H.); wangshengyu@muc.edu.cn (S.W.); 2 Beijing Key Lab of Traditional Chinese Medicine Collateral Disease Theory Research, School of Traditional Chinese Medicine, Capital Medical University, Beijing 100069, China; maruonan@mail.ccmu.edu.cn (R.M.); zhaohao@mail.ccmu.edu.cn (H.Z.); zhaoxiaoyi@mail.ccmu.edu.cn (X.Z.); wangxing@ccmu.edu.cn (X.W.)

**Keywords:** bitter taste receptor, GNAT3, NF-κB, inflammation, BEAS-2B

## Abstract

This study evaluates and compares the protective effects of several type II taste receptor (T2R) agonists against LPS (lipopolysaccharide)-induced inflammatory damage in BEAS-2B cells, focusing on their action via an α-gustducin (encoded by *GNAT3*)-dependent signaling pathway that leads to NF-κB inhibition. To investigate gene expression, mRNA levels of target inflammatory cytokines and T2R subtypes were quantified by qRT-PCR. Cytotoxicity assessment of LPS and bitter agonists was conducted using the CCK-8 assay. The activation status of the NF-κB pathway was examined by Western blot analysis of total and phosphorylated forms of p65 and IκB. Finally, the specific and essential role of GNAT3 was definitively validated through siRNA-mediated gene knockdown. LPS treatment induced significant upregulation of IL-6 and IL-8 mRNA, along with increased phosphorylation of p65 and IκB in BEAS-2B cells. A direct, parallel comparison of the bitter taste agonists PTC (phenylthiourea), QN (quinine), CPD (carisoprodol), and LK (chloroquine) revealed their capacity to upregulate specific T2R subtypes, suppressing inflammatory mediator release and NF-κB activation. Critically, upon *GNAT3* silencing, the inhibitory effects of all tested agonists on p-p65/p65 and p-IκB/IκB ratios were significantly attenuated, without altering total p65 or IκB abundance. This provides direct genetic evidence that GNAT3 is specifically required for mediating the anti-inflammatory effects elicited by these T2R agonists. Multiple bitter receptor agonists exert anti-inflammatory effects on airway epithelial cells in a GNAT3-dependent manner. Our study advances the field by systematically comparing agonist efficacy and establishing the indispensable role of GNAT3 within the anti-inflammatory signaling cascade triggered by T2R agonists, thereby revealing a refined mechanistic insight and potential therapeutic target for inflammatory lung diseases.

## 1. Introduction

Inflammation is a fundamental pathological process primarily characterized by defensive reactions, occurring in living tissues with vascular systems in response to various injurious stimuli. Pneumonia is a common respiratory infection primarily caused by bacteria, viruses, fungi, or parasites, exhibiting high incidence and mortality rates globally [1]. Notably, diseases such as sepsis and acute respiratory distress syndrome (ARDS) secondary to pneumonia also remain associated with substantial mortality. Currently, pharmacotherapy for pneumonia primarily includes antibiotics, antiviral agents, antifungal drugs, glucocorticoids, and other medications. While continuous advancements have been achieved in the pharmacotherapeutic management of pneumonia, considerable challenges persist, such as the development of drug resistance, adverse drug reactions, limited therapeutic options for specific pathogens, and the requirement for optimized medication strategies. Therefore, identifying novel therapeutic targets, exploring and developing drugs through new mechanisms and pathways, or investigating combination therapies represent promising research directions to address the current limitations of pharmacotherapy for pneumonia [2,3].

T2Rs are a class of G protein-coupled receptors (GPCRs) within the taste system, primarily responsible for detecting bitter tastes and assisting the body in identifying potentially harmful substances. In humans, T2Rs are also expressed in non-taste tissues such as the lungs, trachea, and alveolar macrophages [4]. Studies indicate that subtypes such as T2R14 and T2R38 are expressed in human airway smooth muscle, while T2R4, T2R7, T2R8, T2R10, T2R43, and T2R46 are distributed in bronchial epithelial cells [5,6]. Activation of these receptors in the lungs can induce relaxation of airway smooth muscle, thereby dilating bronchi and alleviating airway obstruction, holding potential therapeutic value for obstructive lung diseases [7]. Additionally, emerging evidence suggests that T2Rs function as sentinels for monitoring environmental challenges and coordinating defensive endocrine, behavioral, and immune responses throughout the body [8]. Critically, the canonical bitter taste signaling component α-gustducin has been identified in specialized chemoreceptive cells of the airway epithelium, where it colocalizes with downstream effector PLCβ2, confirming the existence of a functional T2R-gustducin signaling axis in the respiratory tract [9].

In LPS-induced pneumonia, the pulmonary microenvironment undergoes a series of changes. Macrophages polarize into proinflammatory and anti-inflammatory phenotypes [10], T lymphocytes secrete proinflammatory factors, neutrophils infiltrate the site of inflammation, and immune cells become activated and undergo functional shifts to regulate local immune responses and tissue homeostasis. During the course of pulmonary inflammation, interactions between stromal cells and immune cells jointly regulate the homeostasis of the pulmonary microenvironment [8].

Pulmonary epithelial cells play a crucial role as stromal cells in both innate and adaptive immunity. During pneumonia infection, these cells undergo inflammatory responses and repair processes while interacting with various immune cells—including macrophages, dendritic cells, T cells, and B cells—to regulate immune responses [11,12]. When invaded by pathogens such as bacteria or viruses, pulmonary epithelial cells secrete antimicrobial peptides (e.g., lactoferrin, lysozyme) and surfactants to directly eliminate pathogens, while simultaneously expelling inhaled microorganisms through ciliary movement [13]. Pulmonary epithelial cells recognize pathogens via Toll-like receptors (TLRs), activating the NF-κB pathway to regulate inflammation. Studies indicate that LPS stimulation activates the IKK complex (IKKα/β/γ), promoting phosphorylation and degradation of IκBα. This releases the p65-p50 heterodimer into the nucleus to initiate target gene transcription. NF-κB activation promotes the release of proinflammatory cytokines such as TNF-α and IL-1β, further amplifying the inflammatory response and recruiting inflammatory cells, leading to tissue damage [14,15]. Following injury, lung epithelial cells release IL-33, which stimulates the ST2 receptor on Treg cells, prompting them to release the bidirectionally regulatory protein. This protein induces mitotic and epithelial cell differentiation signals, synergistically regulating the post-injury repair process [16].

In recent years, research on the role of bitter taste receptors in pulmonary inflammation has made significant progress. Targeted modulation of bitter taste receptors may offer novel therapeutic strategies for inflammatory lung diseases. Previous studies have demonstrated that phenylthiourea (PTC, a T2R38 agonist) attenuates LPS-induced injury in human pulmonary large vessel endothelial cells [17]. Additionally, quinine (QN, an agonist for T2R4, T2R7, T2R10, T2R14, T2R31, T2R39, T2R40, T2R43, T2R44, T2R46 agonist), and carisoprodol (CPD, T2R14 agonist) can suppress inflammatory factor production in macrophages [18]; chloroquine (LK, T2R3, T2R7, T2R10, T2R39 agonist) and its derivatives exhibit broad-spectrum antiviral and anti-inflammatory properties [15]. However, the precise mechanisms by which different T2R subtype agonists (e.g., PTC, QN, CPD, and LK) regulate inflammatory responses through lung epithelial cells remain to be further elucidated.

Therefore, this study was designed to systematically evaluate and compare the anti-inflammatory effects of four distinct T2R agonists in LPS-challenged human bronchial epithelial cells, and to definitively determine the specific requirement of GNAT3 in mediating these agonist-elicited effects, thereby elucidating the indispensable role of GNAT3 within this anti-inflammatory signaling cascade.

## 2. Results

### 2.1. LPS Promotes the Expression Levels of Inflammatory Cytokines in BEAS-2B Cells

**Figure 1:**
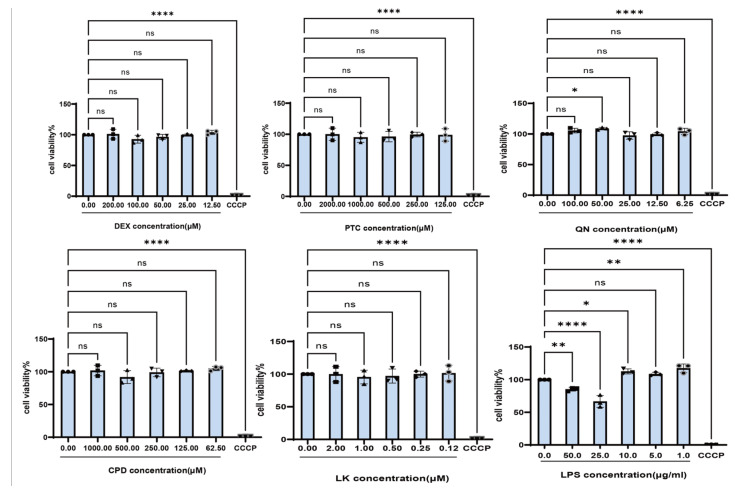
Effects of Different Concentrations of DEX, PTC, Quinine, CPD, LK, and LPS on the Viability of BEAS-2B Cells. ns, not significant, * *p* < 0.05, ** *p*< 0.01, **** *p* < 0.0001 compared with the control group. n = 3.

To investigate the protective effects of bitter taste receptor agonists on pulmonary epithelial cells, the safe concentration ranges for each agonist were first determined, and an LPS-induced BEAS-2B cell injury model was successfully established. Based on the in vitro culture of BEAS-2B, cell viability after treatment with different concentrations of LPS was assessed using the CCK-8 assay. Results showed that 200.0 µM DEX, 2.0 mM PTC, 0.1 mM QN, 1.0 mM CPD, and 2.0 µM LK exhibited no significant toxicity toward BEAS-2B cells within 24 h (Figure 1). These concentrations were therefore selected for subsequent experiments.

**Figure 2:**
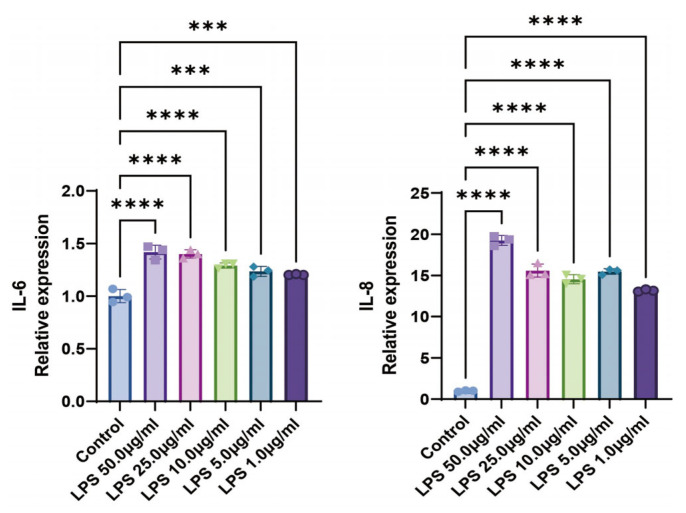
LPS at Different Concentrations Induces IL-6 and IL-8 mRNA Expression in BEAS-2B Cells. *** *p* < 0.001, **** *p* < 0.0001 compared with control group. n = 3.

CCK-8 assays demonstrated that LPS showed no cytotoxicity in BEAS-2B cells below 25.0 µg/mL within 22 h. Stimulation with various LPS concentrations significantly elevated IL-6 and IL-8 mRNA expression levels in cells (Figure 2). To maintain normal cellular viability for subsequent experiments, a final cell injury model condition of 1.0 µg/mL LPS treatment for 22 h was selected for further investigation. Representative images illustrating the corresponding cellular morphology are provided in Supplementary Figure S1.

### 2.2. Bitter Receptor Agonists Specifically Upregulate Bitter Receptor Expression

**Figure 3:**
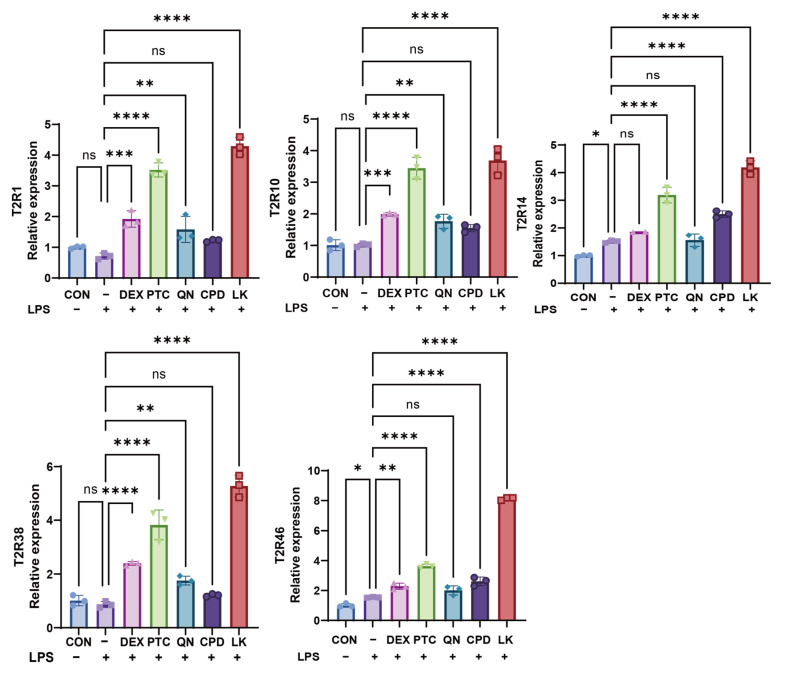
Effects of DEX (0.1 mM), PTC (1.0 mM), QN (50.0 µM), CPD (0.5 mM), and LK (1.0 µM) on LPS (1.0 μg/mL)-Induced mRNA Expression Levels of T2R1, T2R10, T2R14, T2R38, and T2R46 in BEAS-2B Cells. ns, not significant, * *p* < 0.05, ** *p*< 0.01, *** *p* < 0.001, **** *p* < 0.0001 compared with the control group. n = 3.

We detected the expression of T2R1/10/14/38/45 mRNA. As shown in Figure 3, compared with the model group (LPS 1.0 μg/mL), each bitter receptor agonist specifically upregulated the mRNA expression of specific T2Rs in BEAS-2B cells. Specifically, PTC (1.0 mM) significantly upregulated T2R1/10/14/38/46, QN (50.0 µM) significantly upregulated T2R1/10/38/46, CPD (0.5 mM) significantly upregulated T2R14/46, and LK (1.0 µM) significantly upregulated T2R1/10/14/38/46 expression within 24 h.

### 2.3. PTC, QN, CPD, and LK Regulate LPS-Induced Inflammatory Cytokine Expression in BEAS-2B Cells via GNAT3

**Figure 4:**
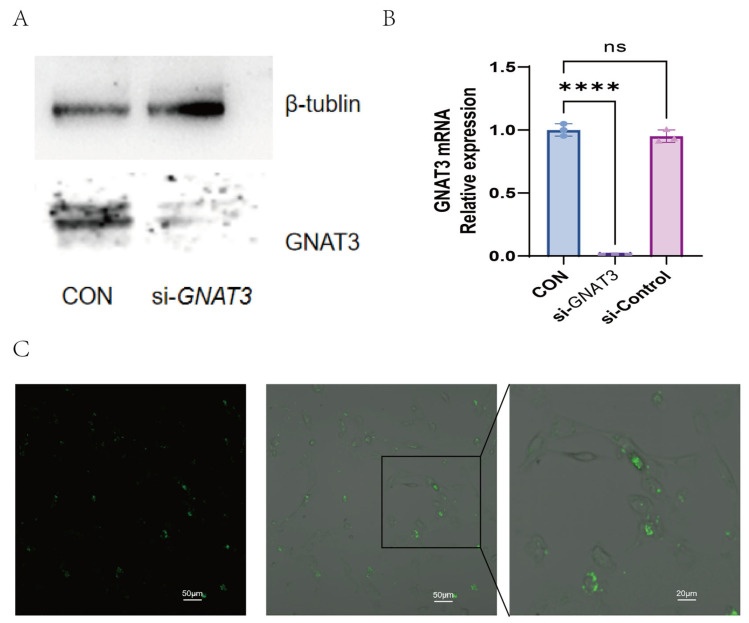
BEAS-2B *GNAT3* siRNA Validation. (**A**) WB bands comparing BEAS-2B *GNAT3* siRNA group with WT group; (**B**) mRNA expression levels in cells after si-*GNAT3* transfection; (**C**) Merged image of green fluorescence (FAM-NC siRNA) and brightfield illumination. Green signal demonstrates successful transfection and intracellular distribution of the non-targeting control siRNA. Scale bars: 50 µm (left and middle panels), 20 µm (right panel). ns, not significant, **** *p* < 0.0001 compared with the control group. n = 3.

**Figure 5:**
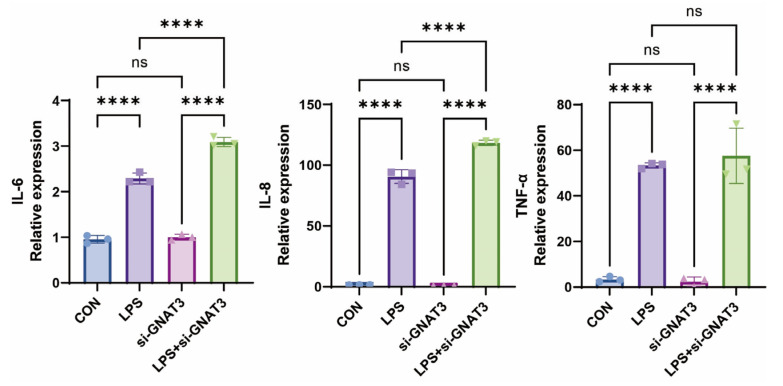
Effects of LPS (1.0 µg/mL) induction on IL-6, IL-8, and TNF-α mRNA levels in WT and si-*GNAT3* BEAS-2B cells. ns, not significant, **** *p* < 0.0001 compared with the control group. n = 3.

To investigate whether bitter receptor agonists exert anti-inflammatory effects via GNAT3, we compared inflammatory factor expression levels in wild-type BEAS-2B cells and si-*GNAT3*-treated BEAS-2B cells following LPS induction. Our initial confirmation of GNAT3 expression in BEAS-2B cells enabled us to directly test its functional necessity. Compared with the normal control group, both GNAT3 mRNA and protein expression levels were significantly reduced in the si-*GNAT3* group (Figure 4). Under 1.0 µg/mL LPS stimulation, IL-6 and IL-8 mRNA expression levels were significantly upregulated in *GNAT3*-silenced BEAS-2B cells compared to WT cells, while no significant changes were observed in the blank control group (Figure 5).

**Figure 6:**
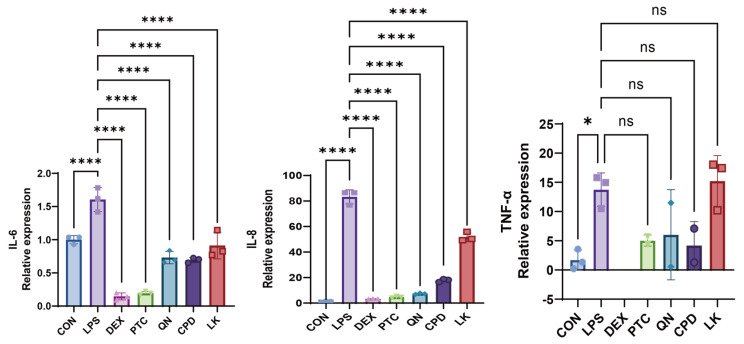
Effects of DEX (0.1 mM), PTC (1.0 mM), QN (50.0 µM), CPD (0.5 mM), and LK (1.0 µM) on IL-6, IL-8, and TNF-α mRNA levels in WT-Type BEAS-2B cells. ns, not significant, * *p* < 0.05, **** *p* < 0.0001 compared with the control group. n = 3.

**Figure 7:**
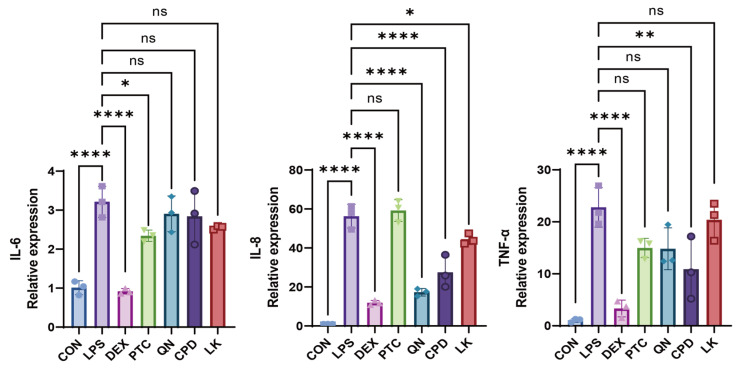
Effects of DEX (0.1 mM), PTC (1.0 mM), QN (50.0 µM), CPD (0.5 mM), and LK (1.0 µM) on IL-6, IL-8, and TNF-α mRNA levels in si-*GNAT3*-transfected BEAS-2B cells. ns, not significant, * *p* < 0.05, ** *p*< 0.01, **** *p* < 0.0001 compared with the control group. n = 3.

In WT BEAS-2B cells, treatment with PTC (1.0 mM), QN (50.0 µM), CPD (0.5 mM), and LK (1.0 µM) significantly inhibited LPS-induced IL-6 and IL-8 mRNA expression; TNF-α expression was too low to yield detectable amplification signals within the specified RT-qPCR cycle threshold (Figure 6, Figures S2A and S4A). In *GNAT3*-silenced BEAS-2B cells, PTC (1.0 mM), significantly downregulated IL-6 expression; QN (50.0 µM), CPD (0.5 mM), and LK (1.0 µM) all significantly downregulated IL-8 expression; additionally, CPD (0.5 mM) significantly inhibited TNF-α expression (Figure 7, Figures S2B and S4B).

**Figure 8:**
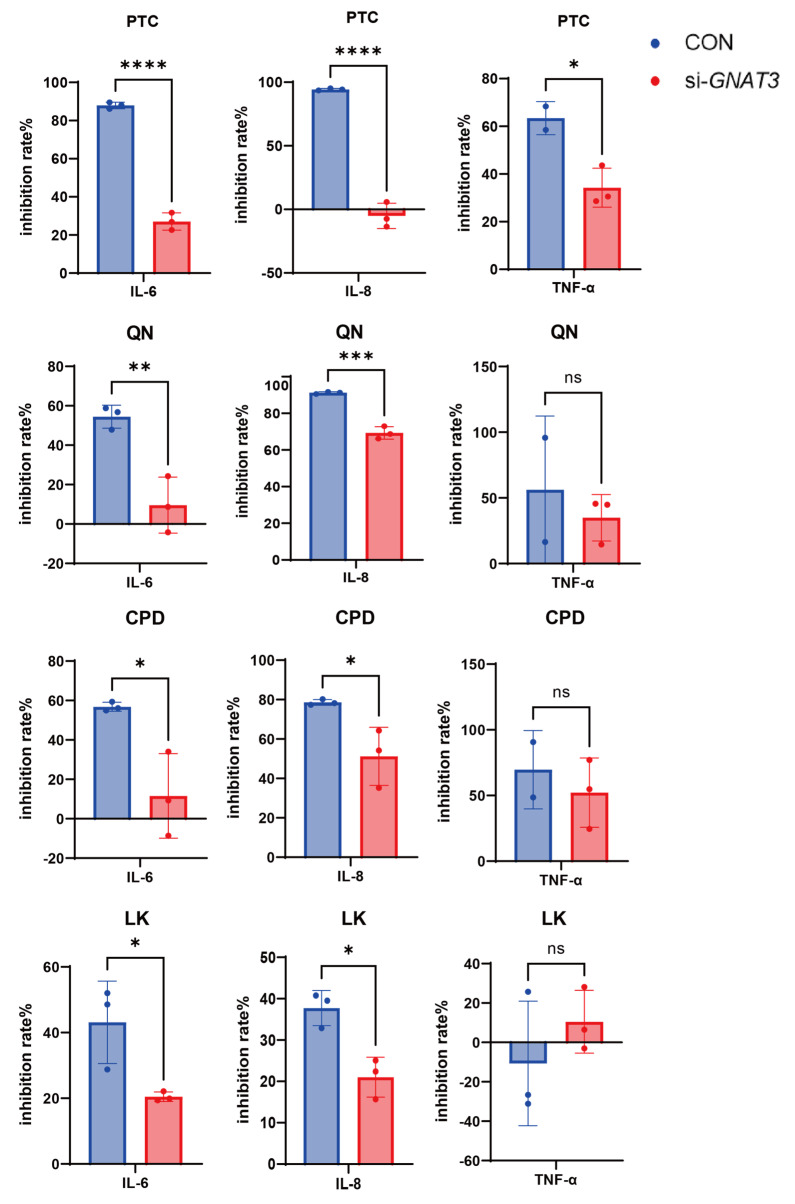
Comparison of LPS-induced IL-6, IL-8, and TNF-α mRNA inhibition rates in WT and si-*GNAT3* BEAS-2B cells by DEX (0.1 mM), PTC (1.0 mM), QN (50.0 µM), CPD (0.5 mM), and LK (1.0 µM). ns, not significant, * *p* < 0.05, ** *p* < 0.01, *** *p* < 0.001, **** *p* < 0.0001 compared with the control group. n = 3.

Compared to WT and si-*GNAT3* BEAS-2B groups, BEAS-2B cells exhibited altered responses to PTC (1.0 mM), QN (50.0 µM), CPD (0.5 mM), and LK (1.0 µM) following *GNAT3* silencing. To quantify this difference, we analyzed the inhibition rates of LPS-induced inflammatory mediators by each drug. Compared to WT cells, si-*GNAT3* cells exhibited reduced inhibition rates of IL-6 and IL-8 under PTC (1.0 mM), QN (50.0 µM), CPD (0.5 mM), and LK (1.0 µM) treatments. Notably, under PTC (1.0 mM) intervention, the inhibition rate of TNF-α was significantly diminished (Figure 8, Figures S3 and S5).

### 2.4. Effects of PTC, QN, CPD, and LK via GNAT3 on LPS-Induced NF-κB Pathway Protein Expression in BEAS-2B Cells

**Figure 9:**
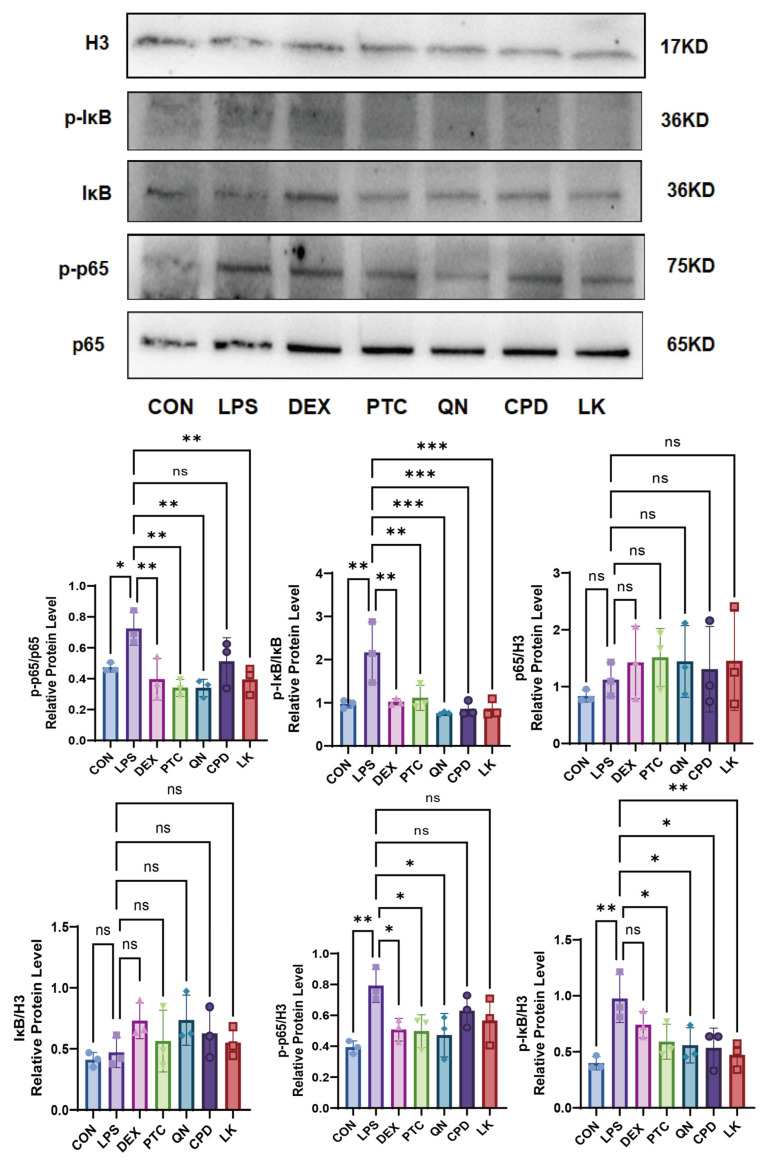
Comparison of the inhibitory effects of DEX (0.1 mM), PTC (1.0 mM), QN (50.0 µM), CPD (0.5 mM), and LK (1.0 µM) on LPS-induced p-p65, p-IκB, p65, and IκB in wild-type BEAS-2B cells. ns, not significant, * *p* < 0.05, ** *p* < 0.01, *** *p* < 0.001. n = 3.

Compared to the control group, LPS treatment significantly upregulated phosphorylation-IκB (p-IκB) and phosphorylation-p65 (p-p65). Concurrently, LPS induced a sharp decrease in total IκB protein levels. The level of total p65 remained relatively stable across all groups. DEX (0.1 mM) effectively reversed the effects of LPS, significantly suppressing the upregulation of p-IκB and p-p65 and preventing IκB degradation. Both PTC (1.0 mM) and CPD (0.5 mM) blocked the LPS-induced production of p-IκB and p-p65 and effectively maintained IκB protein levels. QN (50 µm) and LK (1 µm) also demonstrated clear inhibitory effects, partially reducing the levels of p-IκB and p-p65 and providing a certain degree of protection against IκB degradation (Figure 9).

**Figure 10:**
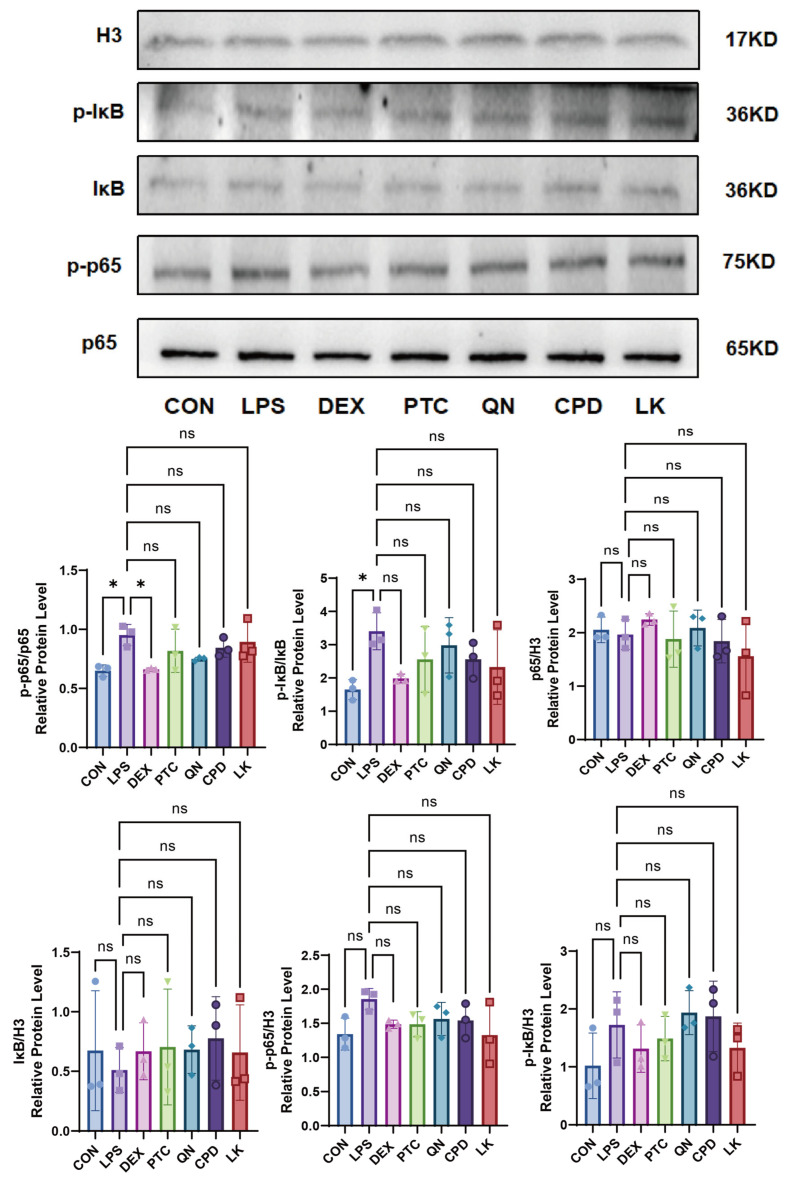
Comparison of the inhibitory effects of DEX (0.1 mM), PTC (1.0 mM), QN (50.0 µM), CPD (0.5 mM), and LK (1.0 µM) on LPS-induced p-p65, p-IκB, p65, and IκB in si-*GNAT3* BEAS-2B cells. ns, not significant, * *p* < 0.05. n = 3.

In the si-*GNAT3* BEAS-2B cell model, LPS treatment significantly upregulated the expression levels of phosphorylated IκB (p-IκB) and phosphorylated p65 (p-p65) compared to the control group, while the levels of total IκB and total p65 proteins remained stable across all groups. DEX (0.1 mM) effectively inhibited LPS-induced upregulation of p-IκB and p-p65. In contrast, PTC (1.0 mM), CPD (0.5 mM), QN (50 µM), and LK (1 µM) did not exhibit significant inhibitory effects (Figure 10).

**Figure 11:**
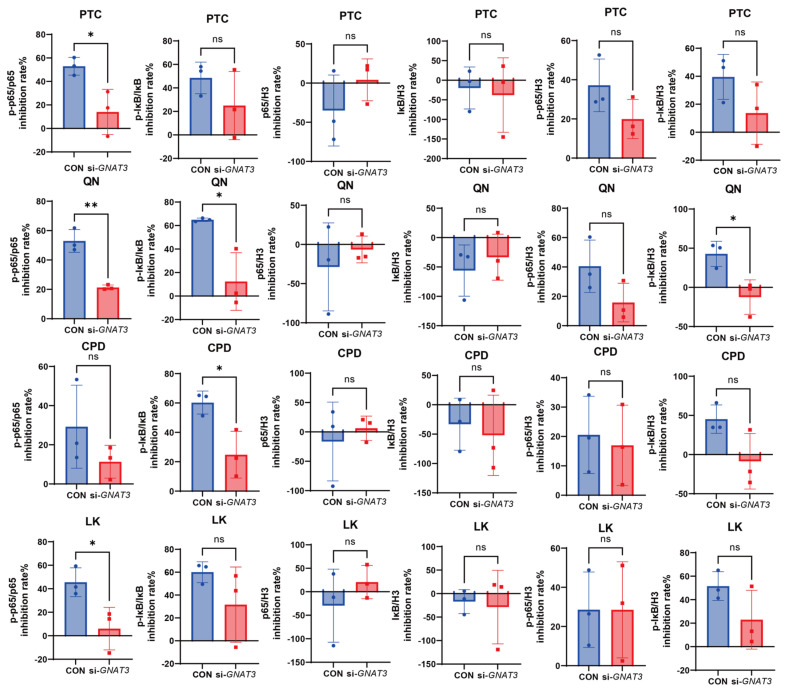
Comparison of LPS-induced p-p65/p65, p-IκB/IκB, p65/H3, IκB/H3, p-p65/H3, and p-IκB/H3 inhibition rates in WT and si-*GNAT3* BEAS-2B cells by DEX (0.1 mM), PTC (1.0 mM), QN (50.0 µM), CPD (0.5 mM), and LK (1.0 µM). ns, not significant, * *p* < 0.05, ** p < 0.01.

Further comparison of WT versus si-*GNAT3* BEAS-2B cells revealed that *GNAT3* silencing significantly altered BEAS-2B cell responses to PTC, QN, CPD, and LK. To quantitatively assess this difference, we analyzed the inhibition rates of each drug on LPS-induced p-p65/p65, p-IκB/IκB, p65/H3, IκB/H3, p-p65/H3, and p-IκB/H3. Results showed that in si-*GNAT3* cells, the inhibition rates of PTC, QN, and LK on p-p65/p65 were significantly lower than in WT cells, while the inhibition rates of QN and CPD on p-IκB/IκB were also significantly reduced. *GNAT3* silencing did not affect total p65 and IκB protein levels, indicating that PTC, QN, CPD, and LK primarily influence the NF-κB signaling pathway by regulating the phosphorylation processes of p65 and IκB, thereby modulating inflammatory cytokine expression (Figure 11).

## 3. Discussion

This study investigated how bitter receptor agonists exert anti-inflammatory effects in a pulmonary epithelial cell inflammation model, specifically via their inhibition of inflammatory mediators (e.g., IL-6, IL-8) through the T2R-gustducin/NF-κB signaling pathway. These findings provide theoretical and experimental support for the development of anti-pneumonia drugs targeting T2Rs. Using human bronchial epithelial cells BEAS-2B as a model, this study investigated the roles of four bitter taste receptor agonists PTC, QN, CPD, and LK in LPS-induced inflammatory responses and their GNAT3-dependent regulatory mechanisms. Results revealed that LPS treatment significantly elevated intracellular p-IκB and p-p65 protein levels while promoting IL-6 and IL-8 mRNA expression, successfully establishing an inflammatory cell model. In wild-type cells, bitter receptor agonists PTC, QN, CPD, and LK specifically upregulated the expression of multiple T2R subtypes in BEAS-2B cells. All agonists effectively suppressed LPS-induced inflammatory factor expression and the phosphorylation of key NF-κB pathway proteins, with PTC and CPD exhibiting stronger inhibitory effects. Subsequent siRNA-mediated silencing of the *GNAT3* gene significantly attenuated the anti-inflammatory response of BEAS-2B cells to these agonists. Specifically, in si-*GNAT3* cells, the inhibition rates of p-p65/p65by PTC, QN, and LK were markedly reduced, while the inhibitory effects of QN and CPD on p-IκB/IκB were also significantly weakened. Notably, *GNAT3* silencing did not affect total p65 and IκB protein levels, indicating that these agonists primarily intervene in the NF-κB pathway by regulating phosphorylation processes rather than altering total protein content.

The partial preservation of anti-inflammatory effects following *GNAT3* knockdown suggests the possible involvement of gustducin-independent pathways. In the canonical model, α-gustducin (encoded by *GNAT3*) serves as the primary Gα protein directly downstream of T2R activation. Therefore, targeting GNAT3 effectively probes the core of this classical signaling axis, and the significant attenuation of agonist effects observed underscores its central role. However, T2Rs, as GPCRs, may exhibit context-dependent coupling to alternative Gα subunits (e.g., Gαi, Gα14/15) or signal through Gβγ complexes independent of a specific Gα, which could account for the residual activity. Furthermore, non-receptor-mediated effects, such as modulation of lysosomal function or induction of endoplasmic reticulum stress by high agonist concentrations, represent plausible alternative mechanisms that warrant future investigation. Thus, while our data establish GNAT3 as a critical and specific mediator within the principal T2R signaling cascade in lung epithelial cells, they also highlight the potential complexity and redundancy of bitter agonist-induced cellular responses.

A primary limitation of this study is the use of relatively high concentrations of T2R agonists in our in vitro model, following precedents in the literature [18,19,20], which may raise concerns regarding potential non-specific effects. However, it is crucial to emphasize that these concentrations were confirmed to be non-cytotoxic, and the core mechanistic finding—that GNAT3 is indispensable for the anti-inflammatory effect—is robustly supported by specific genetic evidence. Moreover, the assessment of the inflammatory cytokines (IL-6, IL-8, TNF-α) relies on mRNA quantification. Future work should incorporate protein-level analyses, such as ELISA, to directly measure cytokine secretion and confirm the functional output of the pathway. Nevertheless, the core conclusions regarding the comparative efficacy of T2R agonists and the essential role of GNAT3 remain robustly supported by protein-level data on NF-κB signaling. Furthermore, while our data demonstrate that T2R agonists upregulate the expression of specific receptor subtypes and that their anti-inflammatory action requires GNAT3, the study does not establish a causal role for any single T2R subtype. Future studies employing siRNA or CRISPR-Cas9 knockout of individual T2Rs are warranted to unequivocally attribute the observed effects to specific receptors and to map agonist-receptor pairings within this pathway.

## 4. Materials and Methods

### 4.1. Cell Culture

Human bronchial epithelial cell line BEAS-2B (Servicebio, Wuhan, China) was cultured in a standard growth medium composed of 89% DMEM high-glucose, 10% fetal bovine serum (FBS), and 1% penicillin-streptomycin (100×) (Servicebio, Wuhan, China, Cat. No. GZ10202) at 37 °C in a humidified incubator with 5% CO_2_. Cells were fed with fresh medium every two days and passaged at a 1:3 split ratio every third day. Cells at passage 5 and passage 6 were used for the qPCR and WB experiments, respectively.

### 4.2. Cell Viability Assay

To assess cell viability, the Cell Counting Kit-8 (CCK-8) assay was employed in this study. Briefly, BEAS-2B cells were seeded at a density of 1 × 10^4^ cells/well in a 96-well plate. After 24 h of culture, cells were treated with different concentrations of LPS (Sigma-Aldrich, St. Louis, MO, USA, Cat. No. 93572-42-0) for 22 h, or with DEX (OriLeaf, Shanghai, China, Cat. No. 2392-39-4), PTC (OriLeaf, Shanghai, China, Cat. No. S60243), QN (OriLeaf, Shanghai, China, Cat. No. S26966), CPD (ACMEC, Shanghai, China, Cat. No. C98311), and LK (OriLeaf, Shanghai, China, Cat. No. S30429) for 24 h. Subsequently, 10 µL of CCK-8 solution (LABLEAD, Beijing, China, Cat. No. CK001) was added to each well, mixed with the medium (total volume 100 µL/well), and incubated at 37 °C for 1.5 h. The absorbance (Abs) of each group was measured at 450 nm wavelength (n = 3). Blank wells contained only medium without cells; untreated cells served as the control group; the CCCP (Carbonyl Cyanide 3-Chlorophenylhydrazone)-treated group was designated as the toxicity control group. Cell viability was calculated using the following formula:

$$\mathrm{Cell}\;\mathrm{viability}\;\left(\%\right)\;=\;\frac{{\mathrm{OD}}_{\mathrm{experimental}\;\mathrm{group}}\;-\;{\mathrm{OD}}_{\mathrm{blank}\;\mathrm{group}}}{{\mathrm{OD}}_{\mathrm{control}\;\mathrm{group}}\;-\;{\mathrm{OD}}_{\mathrm{blank}\;\mathrm{group}}}\;\times \;100\%$$

### 4.3. Reverse Transcription Quantitative PCR

For RNA extraction and qPCR analysis, BEAS-2B cells were seeded in 6-well plates at a density of 5 × 10^5^ cells/well and allowed to adhere for 24 h. Cells were then subjected to the following treatments: stimulation with LPS (1.0 µg/mL) for 22 h, or pre-treatment with T2R agonists (200.0 µM DEX, 2.0 mM PTC, 0.1 mM QN, 1.0 mM CPD, or 2.0 µM LK) for 2 h prior to, and then concurrently with, LPS for an additional 22 h (resulting in a total agonist exposure of 24 h). Following treatment, total RNA was isolated using the SteadyPure Universal RNA Extraction Kit (Accurate Biology, Guangzhou, China, Cat. No. AG21017) according to the manufacturer‘s instructions. cDNA was synthesized from equal amounts of RNA using the Evo M-MLV RT Premix for qPCR (Accurate Biology, Guangzhou, China, Cat. No. AG11706). Quantitative real-time PCR (qRT-PCR) was subsequently performed using the SYBR Green Premix Pro Taq HS qPCR Kit (Accurate Biology, Guangzhou, China, Cat. No. AG11701) on the Real-Time PCR System (Roche LightCycler 96). The primer sequences used are listed in Table 1.

### 4.4. Western Blot

BEAS-2B cells were seeded at a density of 5 × 10^5^ cells/well in a 6-well plate. After 24 h of culture, cells were treated with different concentrations of 1.0 µg/mL LPS for 0.5 h, or with 200.0 µM DEX, 2.0 mM PTC, 0.1 mM QN, 1.0 mM CPD, and 2.0 µM LK for 2.5 h.

Assessment of *GNAT3* Knockdown Efficiency: For this specific purpose, we prepared total cellular lysates from a parallel set of treated cells. The expression level of α-gustducin (encoded by *GNAT3*) in these total lysates was analyzed by Western blot and normalized to β-tubulin, serving as a robust loading control for total protein. This approach directly and reliably confirms the efficacy of siRNA-mediated *GNAT3* silencing at the protein level.

Analysis of NF-κB Signaling Pathway Activation: To specifically investigate the nuclear translocation and activation of NF-κB, we prepared subcellular fractions from another set of identically treated cells using a commercial Nuclear and Cytoplasmic Protein Extraction Kit (Beyotime, Shanghai, China, Cat. No. P0027). The nuclear protein fraction was used to analyze the levels and phosphorylation status of p65 and IκB. The loading and integrity of this nuclear fraction were monitored by normalizing these target signals to Histone H3, a canonical nuclear marker and loading control.

Total protein concentration in each sample group was determined using the BCA Protein Quantification Kit (LABLEAD, Beijing, China, Cat. No. B5001). Equal amounts of protein (A total of 20 µg of protein was loaded per lane) were separated by 10% SDS-PAGE gel electrophoresis and transferred to PVDF membranes. Membranes were blocked with TBST containing 5% BSA for 2 h, followed by overnight incubation with primary antibodies at 4 °C. Primary antibodies used included: anti-GANT3 (1:1000, Abmart, Shanghai, China, Cat. No. PS09064S), anti-p65 (1:5000, Proteintech, Wuhan, China, Cat. No. 80979-1-RR), anti-p-p65 (1:2000, Proteintech, Wuhan, China, Cat. No. 82335-1-RR), anti-IκBα (1:5000, Proteintech, Wuhan, China, Cat. No. 10268-1-AP), anti-p-IκBα (1:1000, Proteintech, Wuhan, China, Cat. No. 82349-1-RR), anti-β-tubulin (1:5000, Proteintech, Wuhan, China, Cat. No. 10094-1-AP), and anti-Histone H3 (1:2000, Proteintech, Wuhan, China, Cat. No. 17168-1-AP). The next day, membranes were washed with TBST and incubated with HRP-labeled goat anti-rabbit IgG secondary antibody (1:5000, LABLEAD, Beijing, China, Cat. No. S0101) at room temperature for 1 h. Finally, gray-scale analysis and quantification of protein bands were performed using ImageJ 1.53t software (U.S. National Institutes of Health, Bethesda, MD, USA).

### 4.5. siRNA Interference Experiment

To investigate the function of GNAT3, we employed RNA interference to downregulate its expression in BEAS-2B cells. siRNA targeting *GNAT3* and negative control siRNA were purchased from GenScript. Human bronchial epithelial cells BEAS-2B were cultured in BEAS-2B-specific medium at 37 °C in a 5% CO_2_ incubator. Twenty-four hours prior to transfection, cells were seeded at a density of 2 × 10^5^ cells per well in a 6-well plate. Cells were cultured in Opti-Medium low-serum medium (EallBio, Beijing, China, Cat. No. 03.18001A) and transfected when confluence reached 60–70%. Dilute Lipofectamine 2000 (Invitrogen, Wuhan, China, Cat. No. 11668027) and siRNA (50.0 nM) in Opti-Medium low-serum medium prior to complex formation. Mix the diluted solutions and incubate for 20 min. Add the complex dropwise to the cell culture medium and gently mix. Replace with complete medium 6 h post-transfection. Collect cells 48 h post-transfection. Total RNA was extracted using the SteadyPure Universal RNA Extraction Kit. cDNA was synthesized from samples across different groups using a cDNA synthesis kit. Relative expression levels of *GNAT3* mRNA were detected by SYBR Green qPCR, with ACTB as the internal control. Concurrently, GNAT3 protein expression levels were assessed by Western Blot analysis 48 h post-transfection.

### 4.6. Immunofluorescence

Seed cells in confocal culture dishes and perform transfection when cell confluence reaches 50–70%. The transfection procedure is as described in Section 4.5. To assess gene silencing efficiency, 48 h post-transfection, fluorescence protein expression was observed under a fluorescence microscope (OLYMPUS Corporation, Tokyo, Japan, IX2-UCB) using a 20× objective lens, with excitation at 520 nm under dark-field conditions.

### 4.7. Statistical Analysis

Data are presented as the mean ± standard error of the mean (SEM) of *n* independent biological replicates, as indicated in the figure legends. Statistical comparisons between two groups were performed using two-tailed, unpaired Student’s t-tests. For comparisons among more than two groups, one-way ANOVA was applied, followed by Dunnett’s multiple comparisons test. ns, not significant, * *p* < 0.05, ** *p* < 0.01, *** *p* < 0.001, **** *p* < 0.0001. Statistical analysis was performed using GraphPad Prism 9.5.1 (GraphPad, San Diego, CA, USA).

## 5. Conclusions

In summary, this study demonstrates that multiple bitter taste receptor agonists—PTC, QN, CPD, and LK—inhibit LPS-induced NF-κB activation and downstream inflammatory cytokine expression in BEAS-2B cells through a GNAT3-dependent signaling pathway. These agonists specifically upregulate the expression of distinct T2R subtypes and suppress the phosphorylation of p65 and IκB, thereby attenuating inflammatory responses in pulmonary epithelial cells. Critically, siRNA-mediated knockdown of *GNAT3* significantly diminished the anti-inflammatory effects of all tested agonists, providing direct genetic evidence for the indispensable role of gustducin in this signaling cascade. Collectively, our findings deepen the understanding of T2R-mediated immunomodulation in airway epithelium and highlight GNAT3 as a potential therapeutic target for inflammatory lung diseases.

While this study establishes a clear GNAT3-dependent mechanism, several important questions remain. The binding specificities of these agonists to individual T2R subtypes and their downstream effector couplings warrant further investigation, potentially through receptor-specific knockout models. Additionally, validation in animal models of pneumonia will be essential to assess the in vivo efficacy and safety of T2R-targeted therapies. Finally, the downstream signaling events mediated by GNAT3, including potential crosstalk with Ca^2+^ or cAMP pathways, represent promising avenues for further mechanistic exploration.

## Figures and Tables

**Table 1:** Oligonucleotide Primer Sequences for qPCR Assays.

	Forward Primer Sequence (5′→3′)	Reverse Primer Sequence (5′→3′)
TNF-α	CTGCCTGCTGCACTTTGGAG	ACATGGGCTACAGGCTTGTCACT
GNAT3	AGAGGACCAACGACAACTTTATG	AGCCGTTTTATTACCTCAGCC
T2R1	CACCCGGCAAATGAGAAACA	GACAGGATAGACAGCAACGC
T2R10	CAGAAGCTCATGTGAAGGCAAT	GGGATAGATGGCTGTGGTTGT
T2R14	ATACCCTTTACTTTGTCCCTGGCA	TGACAGTGTGCTGCATCTTCT
T2R38	GGGTGATGGTTTGTGTTGGG	CTTGTGGTCGGCTCTTACCT
T2R46	AGTTCCCTTCACTCTGACCCT	GTGGACCTTCATGCTGGGAT
IL-6	ACTCACCTCTTCAGAACGAATTG	CCATCTTTGGAAGGTTCAGGTTG
IL-8	CACCGGAAGGAACCATCTCA	TTGGGGTGGAAAGGTTTGGA
ACTB	TGGCACCCAGCACAATGAA	CTAAGTCATAGTCCGCCTAGAAGCA

## Data Availability

The original contributions presented in this study are included in the article. Further inquiries can be directed to the corresponding authors.

## Supplementary Materials

The following supporting information can be downloaded at: https://www.mdpi.com/article/10.3390/ijms27020997/s1.

## Abbreviations

The following abbreviations are used in this manuscript:

T2R	type 2 bitter taste receptor
LPS	Lipopolysaccharide
DEX	Dexamethasone
PTC	Phenylthiourea
QN	Quinine
CPD	Carisoprodol
LK	Chloroquine
