# Supplementary material for: Defining the Critical Role of α-Gustducin for NF-κB Inhibition and Anti-Inflammatory Signal Transduction by Bitter Agonists in Lung Epithelium
Source: Int J Mol Sci. 2026 Jan 19;27(2):997. doi: 10.3390/ijms27020997 (PMC12842234; doi:10.3390/ijms27020997)
Supplement: Supplementary file 1 [file ijms-27-00997-s001.zip › ijms-4085929-supplementary.pdf]

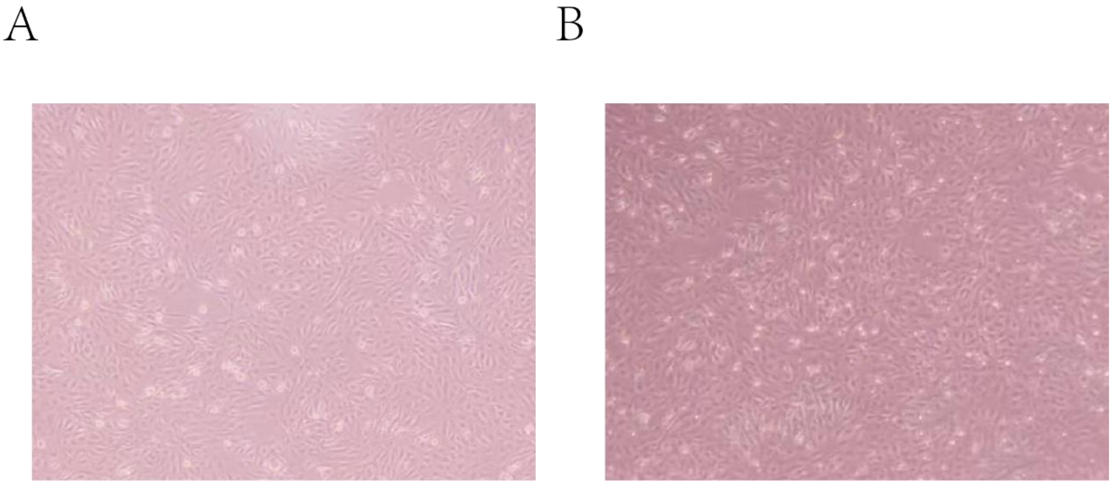

Figure S1. A. BEAS-2B cells under normal culture conditions for 24 hours. B. BEAS-2B cells cultured normally for 2 hours, followed by LPS (1.0 μg/mL) treatment for an additional 22 hours.

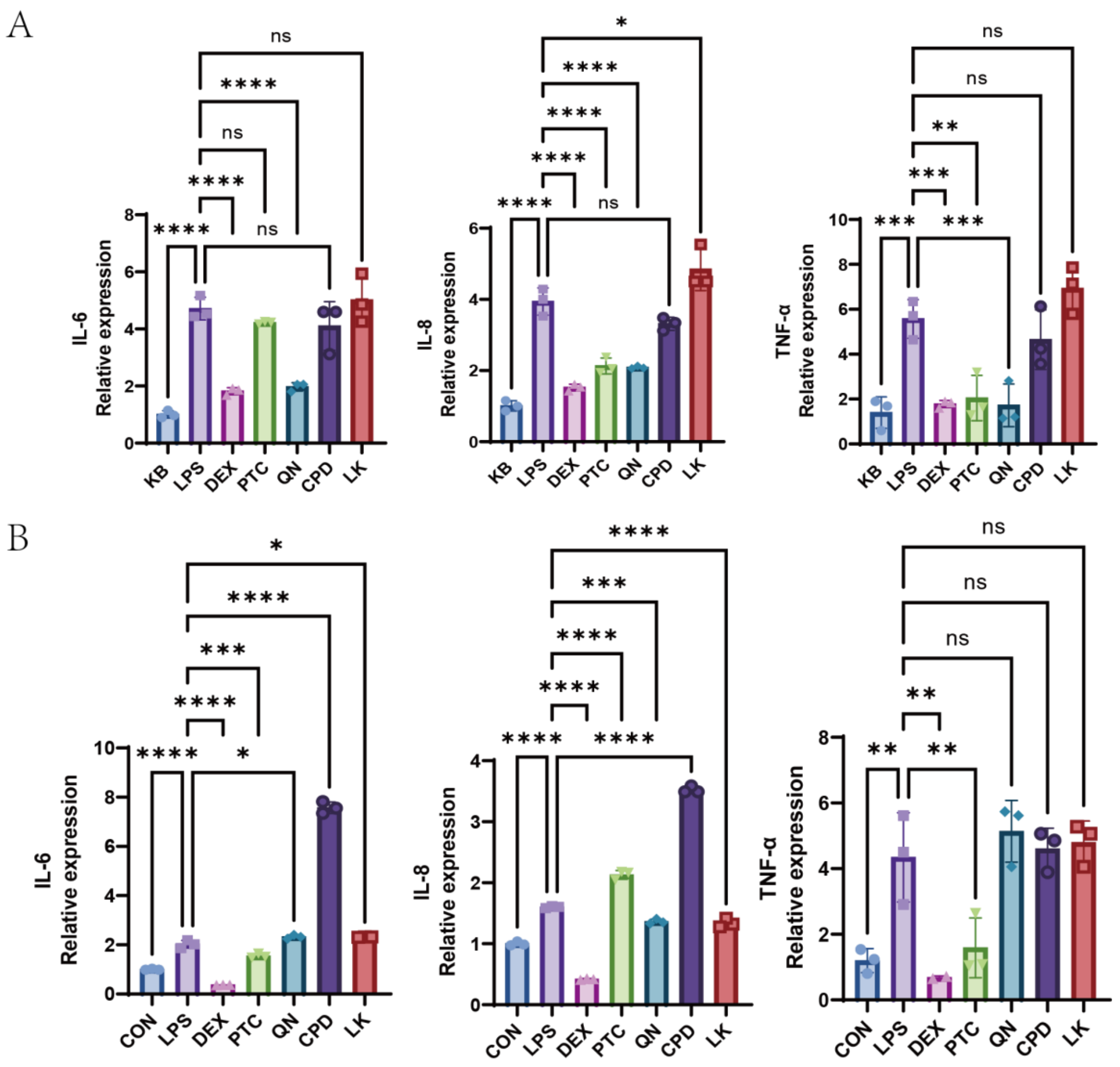

Figure S2. A. Effects of DEX (0.1 mM), PTC (1.0 mM), QN (50.0 μM), CPD (0.5 mM), and LK (1.0 μM) on IL-6, IL-8, and TNF-*α* mRNA levels in WT-Type BEAS-2B cells. B. Effects of DEX (0.1 mM), PTC (1.0 mM), QN (50.0 μM), CPD (0.5 mM), and LK (1.0 μM) on IL-6, IL-8, and TNF-*α* mRNA levels in si-GNAT3 transfected BEAS-2B cells. ns, not significant, *$p < 0.05$, **$p < 0.01$, ***$p < 0.001$, ****$p < 0.0001$ compared with the control group.

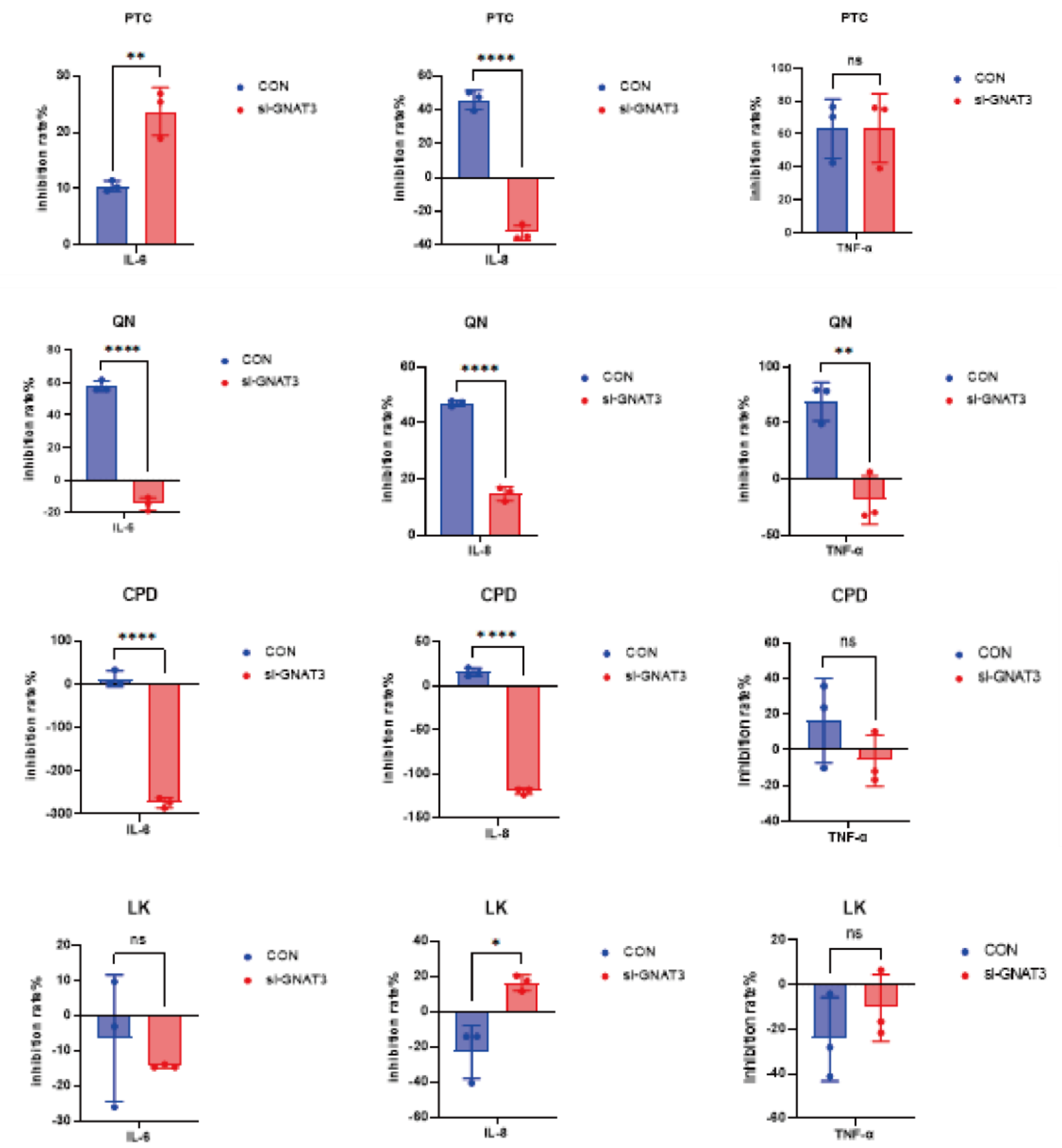

Figure S3. Comparison of LPS-induced IL-6, IL-8, and TNF-α mRNA inhibition rates in WT and si-GNAT3 BEAS-2B cells by DEX (0.1 mM), PTC (1.0 mM), QN (50.0 μM), CPD (0.5 mM), and LK (1.0 μM). ns, not significant, *$p < 0.05$, **$p < 0.01$, ****$p < 0.0001$ compared with the control group.

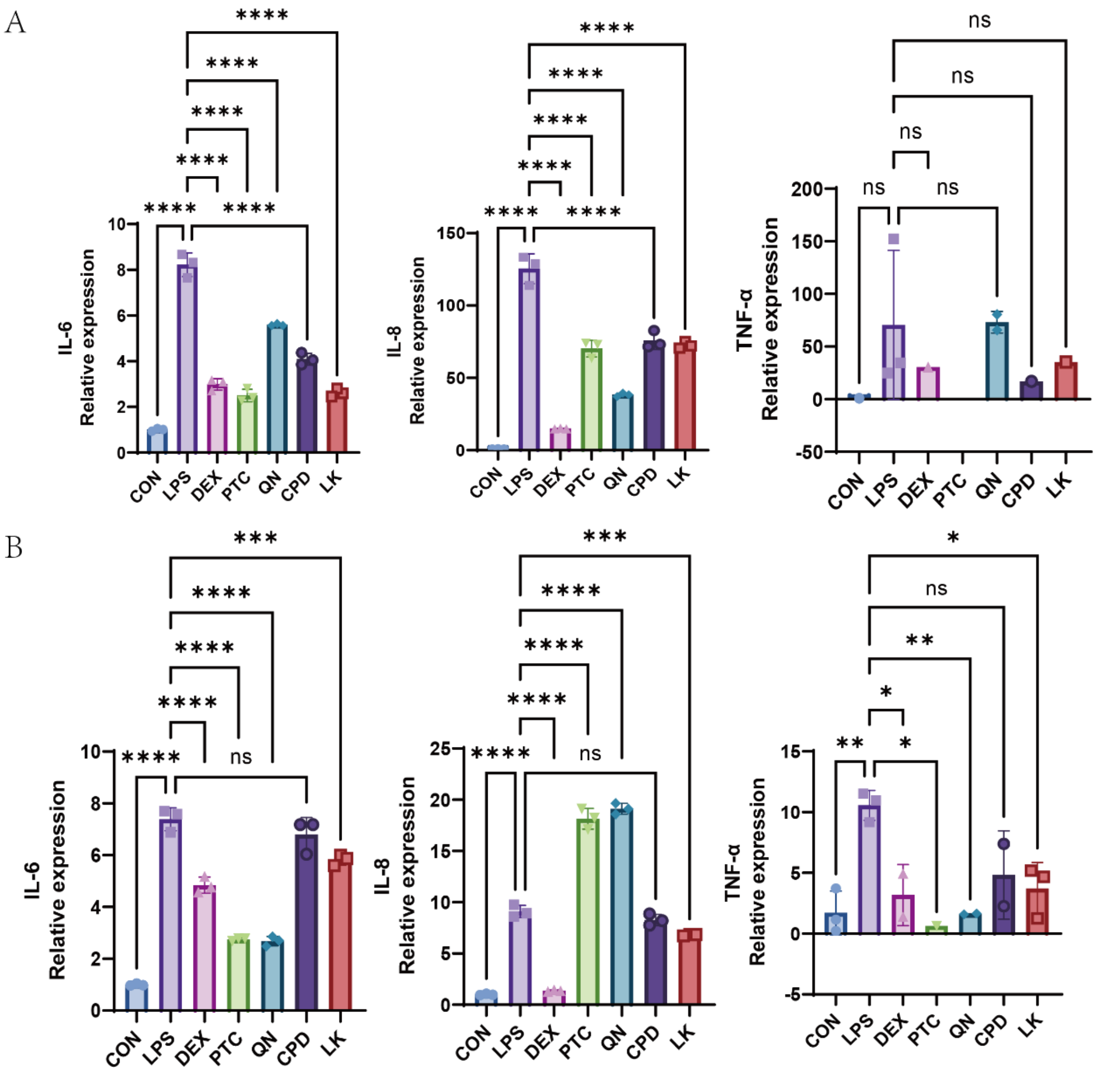

Figure S4. A. Effects of DEX (0.1 mM), PTC (1.0 mM), QN (50.0 μM), CPD (0.5 mM), and LK (1.0 μM) on IL-6, IL-8, and TNF-*α* mRNA levels in WT-Type BEAS-2B cells. B. Effects of DEX (0.1 mM), PTC (1.0 mM), QN (50.0 μM), CPD (0.5 mM), and LK (1.0 μM) on IL-6, IL-8, and TNF-*α* mRNA levels in si-GNAT3 transfected BEAS-2B cells. ns, not significant, $*p < 0.05$, $**p < 0.01$, $****p < 0.0001$ compared with the control group.

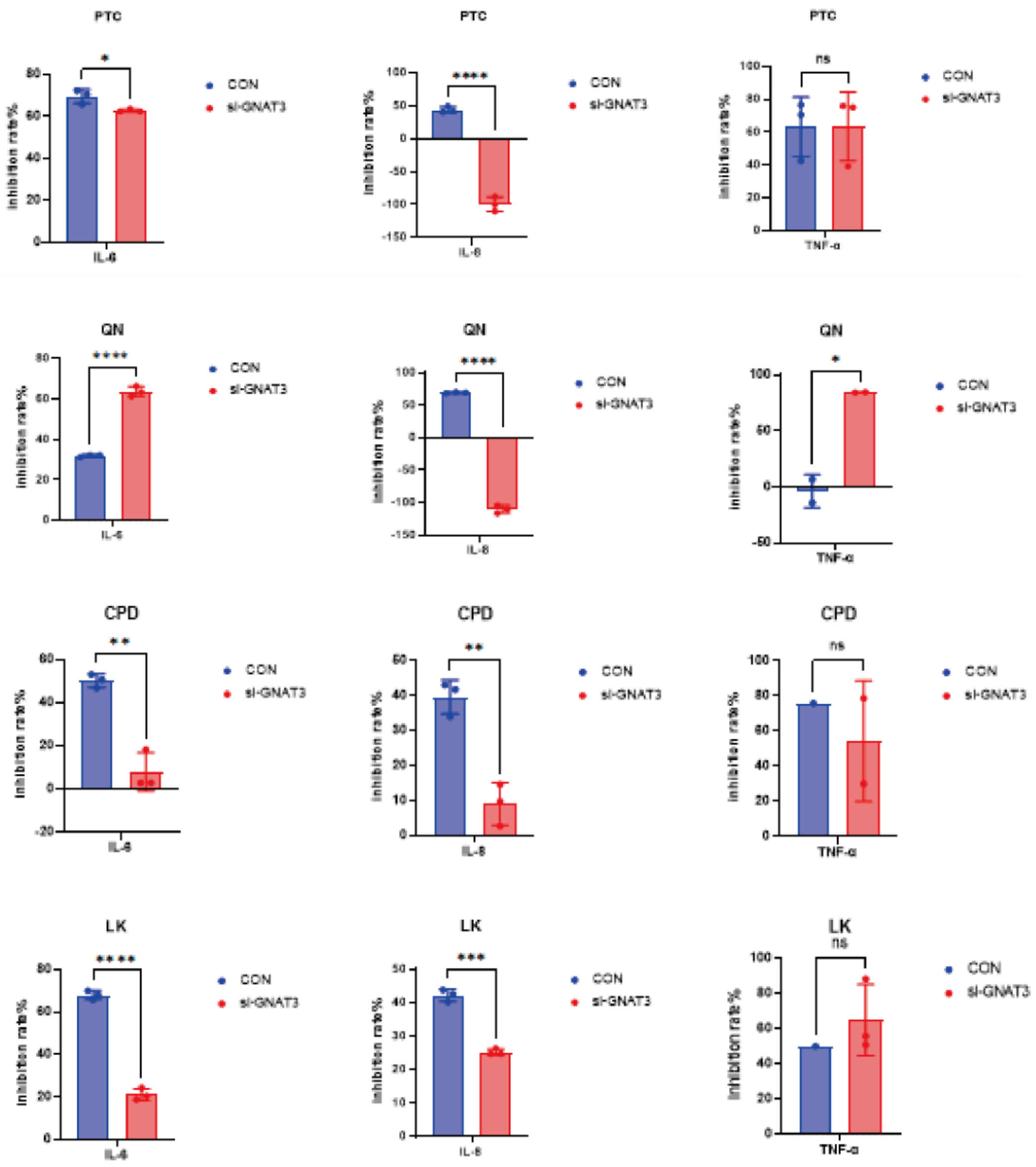

Figure S5. Comparison of LPS-induced IL-6, IL-8, and TNF-α mRNA inhibition rates in WT and si-GNAT3 BEAS-2B cells by DEX (0.1 mM), PTC (1.0 mM), QN (50.0 μM), CPD (0.5 mM), and LK (1.0 μM). ns, not significant, *$p < 0.05$, **$p < 0.01$, ***$p < 0.001$, ****$p < 0.0001$ compared with the control group.
